# Ectopic Expression *of Nolz-1* in Neural Progenitors Promotes Cell Cycle Exit/Premature Neuronal Differentiation Accompanying with Abnormal Apoptosis in the Developing Mouse Telencephalon

**DOI:** 10.1371/journal.pone.0074975

**Published:** 2013-09-20

**Authors:** Sunny Li-Yun Chang, Shih-Yun Chen, Huai-Huei Huang, Hsin-An Ko, Pei-Tsen Liu, Ya-Chi Liu, Ping-Hau Chen, Fu-Chin Liu

**Affiliations:** 1 Institute of Neuroscience, National Yang-Ming University, Taipei, Taiwan, Republic of China; 2 Graduate Institute of Basic Medical Science, China Medical University, Taichung, Taiwan, Republic of China; 3 Graduate Institute of Molecular Systems Biomedicine, China Medical University, Taichung, Taiwan, Republic of China; 4 School of Pharmacy Undergraduate Program, China Medical University, Taichung, Taiwan, Republic of China; Academia Sinica, Taiwan

## Abstract

*Nolz-1*, as a murine member of the NET zinc-finger protein family, is expressed in post-mitotic differentiating neurons of striatum during development. To explore the function of *Nolz-1* in regulating the neurogenesis of forebrain, we studied the effects of ectopic expression of *Nolz-1* in neural progenitors. We generated the Cre-loxP dependent conditional transgenic mice in which *Nolz-1* was ectopically expressed in proliferative neural progenitors. Ectopic expression of *Nolz-1* in neural progenitors by intercrossing the *Nolz-1* conditional transgenic mice with the nestin-Cre mice resulted in hypoplasia of telencephalon in double transgenic mice. Decreased proliferation of neural progenitor cells were found in the telencephalon, as evidenced by the reduction of BrdU−, Ki67− and phospho-histone 3-positive cells in E11.5–12.5 germinal zone of telencephalon. Transgenic *Nolz-1* also promoted cell cycle exit and as a consequence might facilitate premature differentiation of progenitors, because TuJ1-positive neurons were ectopically found in the ventricular zone and there was a general increase of TuJ1 immunoreactivity in the telencephalon. Moreover, clusters of strong TuJ1-expressing neurons were present in E12.5 germinal zone. Some of these strong TuJ1-positive clusters, however, contained apoptotic condensed DNA, suggesting that inappropriate premature differentiation may lead to abnormal apoptosis in some progenitor cells. Consistent with the transgenic mouse analysis *in vivo*, similar effects of *Nozl-1* over-expression in induction of apoptosis, inhibition of cell proliferation and promotion of neuronal differentiation were also observed in three different N18, ST14A and N2A neural cell lines *in vitro.* Taken together, our study indicates that ectopic expression of *Nolz-1* in neural progenitors promotes cell cycle exit/premature neuronal differentiation and induces abnormal apoptosis in the developing telencephalon.

## Introduction

The *nocA* like C_2_H_2_ zinc-finger gene-1 (*Nolz-1*), which is also known as *Zfp503/Znf503* (Mouse Genome Informatics), is previously identified as a murine member of the *noc/Nlz-elbow-tlp-1* (NET) zinc-finger protein family [1]. The NET gene family has been shown to involve in control of a variety of developmental events. The *C. elegans* homologue *Tlp1* regulates asymmetric cell fates of T blast cells [2]. The Drosophila homologues *elB* and *noc* specify the identity of dorsal-ventral branches of trachea [3]. The zebrafish homologue *Nlz1* and *Nlz2* regulate the rhombomere identity in developing hindbrain and the closure of optic fissure in zebrafish eye [4]–[8]. The chick homologue *Nolz-1* controls the subtype identity of motor neurons in developing spinal cord [9]. Recent study shows that mouse *Nolz-1* regulates the neurogenesis in neurosphere culture *in vitro* [10].

*Nolz-1* expression is developmentally regulated in different mouse organs during embryogenesis [9], [11], [12]. In the developing telencephalon, *Nolz-1* is preferentially expressed at high levels in the lateral ganglionic eminence (LGE, the primordium of striatum), and *Nolz-1* expression is gradually down-regulated in the striatum after birth [10], [11]. During striatal neurogenesis, neural progenitor cells reside in the ventricular zone (VZ) and the subventricular zone (SVZ). Upon differentiation, post-mitotic differentiating neurons migrate from the SVZ into the mantle zone (MZ) where differentiating neurons undergo terminal differentiation [13]. Therefore, unlike the VZ which contains proliferative neural progenitors, the SVZ contains both proliferative progenitors as well as early differentiating post-mitotic neurons [13]. It is of interest that *Nolz-1* is expressed at high levels in the SVZ of LGE, but not in the VZ. *Nolz-1* expression level is lower in the differentiated MZ. Moreover, *Nolz-1* is not expressed in Ki67-positive proliferating progenitor cells but is co-expressed with the early neuronal differentiation markers of TuJ1 and Isl-1 indicating that *Nolz-1* is expressed in early post-mitotic striatal neurons [10], [11], [14]. *Nolz-1* therefore serves as a developmental marker for differentiating striatal neurons.

Because *Nolz-1* is not expressed by proliferative neural progenitors in the germinal zone [11], we investigated the effects of ectopic expression of *Nolz-1* in neural progenitors. We generated the *Nolz-1* conditional transgenic mice using the Cre/loxP-mediated DNA recombination technology [15]. Ectopic expression of *Nolz-1* in neural progenitors was achieved by intercrossing the *Nolz-1* conditional transgenic mice with the nestin-Cre driver mice [16], [17]. Telencephalic hypoplasia was found in the resulting double transgenic mice. Further examination showed that transgenic expression of *Nolz-1* led to decreased proliferation and precocious differentiation of progenitor cells in developing telencephalon. Moreover, aberrant differentiation induced by transgenic *Nolz-1* appeared to result in abnormal cell death. These findings suggest a role of *Nolz-1* in promoting neuronal differentiation during neurogenesis in developing telencephalon.

## Methods

### Generation of Transgenic Mice

The transgenic plasmid of pLacZ-Nolz-1-eGFP (pZNG), for generating LacZ^floxed^Nolz-1^eGFP^ transgenic mice, was constructed using the pCCALL2 plasmid (Kindly provided by Dr. A. *Nagy*, University of Toronto) as backbone [18]. The coding sequence of C-terminal Flag-tagged *Nolz-1* was generated by PCR, confirmed by DNA sequencing and then ligated with the downstream IRES-eGFP-polyA cassette which was composed by three DNA fragments released from pNTR-LacZPGKneolox, pCAGGS-eGFP and PGK-puro-lox2a plasmids (kindly provided by Dr. T.-F. Tsai, National Yung-Ming University), respectively. A direct repeat of a 1.2-kb fragment containing the DNase I-hypersensitivesite 4 of the chicken *β*-globin locus (HS4) fragment was released from SalI and XbaI sites of pJC13-1-5′HS4 (kindly provided by Dr. T.-F. Tsai), and was then ligated downstream of the IRES-eGFP-polyA cassette in a reverse orientation to relieve *position silence effects* at *transgene* [19], [20]. The transgenic fragment was released by ScaI and XhoI sites for C57/BL6 pronuclei microinjection (Level Biotechnology, Taipei, Taiwan). Potential founder mice were screened by PCR using tail DNA as template and HS4 specific primers (HS4-51∶5′-CCTCCTTGGGCAACCTGTTCAG-3′; HS4-31∶5′-ATGTGGCACTGAGGGACATGGC-3′). The PCR-positive transgenic lines were further confirmed by Southern blotting. Tail genomic DNAs from LacZ^floxed^Nolz-1^eGFP^ F1 offspring was digested with EcoRI, and the DNA probe containing the eGFP coding region was used in Southern blotting.

### Ethics Statement

The transgenic mice were maintained in the Animal Centers of National Yang-Ming University (NYMU) and China Medical University (CMU). The animal protocols were approved by the Animal Care and Use Committees of NYMU and CMU.

### Mice Breeding and Genotyping

Heterozygous transgenic mice were maintained in C57/BL6 background. Heterozygous LacZ^floxed^Nolz-1^eGFP^ (T) transgenic mice were intercrossed with heterozygous nestin-Cre (nC) mice [16] to obtain nCT, nC, T and wild type (WT) embryos for phenotypic analysis. Genotypes of the transgenic embryos were analyzed by PCR using HS4-51, HS4-31 and Cre specific primers (Cre-F: 5′-TCCAATTTACTGACCGTACAC-3′; Cre-R: 5′-CGCCGTAAATCAATCAATCGATGAG -3′). The day of positivity of vaginal plug was defined as E0.5. At least three litters were analyzed per condition. For BrdU incorporation and the cell cycle exit assays, pregnant mice were i.p. injected with BrdU 1 hr (100 mg/kg) and 4 hr (50 mg/kg), respectively, before being scarified.

### Western Blotting

The cortical tissues, LGE/striatal and MGE tissues of nCT, nC and T littermate brains (E12.5–E13.5 for TuJ1 expression analysis; E16.5 for *tNolz-1* expression analysis) and the cell lysates were collected 24 hr (N18 cells) and 72 hr (N2A cells) after transfection and lyzed in RIPA lysis buffer (20 mM HEPES, pH 7.8, 150 mM NaCl, 1 mM EDTA, 0.1% Triton X-100, 50 mM NaF, 1 mM dithiothreitol, protease inhibitor cocktail). 30–50 µg of total protein of each sample was loaded to SDS-polyacrylamid gel for electrophoresis and transferred to polyvinylidene difluoride (PVDF; Amersham Pharmacia Biotech., Piscataway, NJ) membranes for Western blotting [21]–[24]. The membranes were cut into 2–3 pieces for detection of different proteins according to the molecular weights of the proteins, Nolz-1 (∼65 kDa), TuJ1 (∼50 kDa), PCNA (∼29 kDa), cleaved caspase 3 (∼17/19 kDa), α-tubulin (∼50 kDa) and β-actin (∼42 kDa) proteins. The primary antibodies were rabbit anti-Nolz-1 (1∶1,000) [14], mouse anti-TuJ1 (1∶10,000, Promega, Madison WI), rabbit anti-cleaved caspase 3 (1∶1,000, Cell Signaling Technology, Danvers, MA), mouse anti-PCNA (1∶1,000, Santa Cruz Biotechnology, Dallas, Texas), mouse anti-α-tubulin (1∶1,000, Sigma Aldrich, St. Louis, Missouri) and mouse anti-β-actin (1∶10,000; Sigma Aldrich). Secondary antibody used was horseradish peroxidase conjugated goat anti-rabbit IgG and goat anti-mouse IgG (1∶10,000; Jackson ImmunoResearch Laboratories, West Grove, PA). Before signal detection using enhanced chemiluminescence ECL detection system (Millipore, Billerica, MA), the PVDF membranes were incubated with Elite ABC mixture (1∶200; Vector laboratories, Burlingame, CA) to amplify the signals for Nolz-1 protein detection.

### Cell Culture and Transfection

HEK293T cells, neuroblastoma N18, N2A cells and ST14A cells were cultured at 37°C in Dulbecco’s modified Eagle’s medium (DMEM, Gibco/Life Technologies, Grand Island, NY) supplemented with 10% fetal bovine serum and 1% penicillin and streptomyocin in a 5% CO_2_ incubator[25]–[28]. The pZNG plasmid was co-transfected with pCre-nls plasmid (1∶1; kindly provided by Dr. T.-F. Tsai) into HEK293T cells by calcium-phosphate precipitation method [25]. For over-expression of *Nolz-1* in cell lines, the Nolz-1 expression plasmids, including pCAG-EYFP-CAG-Nolz-1 (pNolz-1) for N18 cells and ST14A cells and pcBIG-Nolz-1 for N2A cells were used. The Nolz-1 expression plasmids and their mock control plasmids were transfected into N18, ST14A and N2A cells by Lipofectamine™ 2000 (Invitrogene/Life Technologies, Grand Island, NY), electroporation (200 V, 5 msec, 1 pulse; BTX ECM 830) and GeneJuice® Transfection Reagent (Novagen, Madison, WI), respectively, following *manufacturers*’ instructions. Results of *Nolz-1* over-expression in HEK293, N18 and ST14A cells were analyzed 24 hr after transfection. For N2A cells, 24 hr after transfection, the culture medium was changed to DMEM with 2% FBS, and the cells were harvested 72 hr after transfection.

### *In situ* Hybridization and Immunostaining

*In situ* hybridization and immunostaining were performed as previously described [11], [20], [29]. The RNA probes included *eGFP* (coding region) and *Nolz-1* (NM145459∶2640-3744). The primary antibodies included rat-anti-BrdU (1∶1,000, Accurate Chemical & Scientific, New York), sheep anti-BrdU (1∶2,000, Biodesign International, Saco, ME), rabbit anti-phospho-histone 3 (PH3) (1∶1,000, Millipore, Billerica, MA), rabbit anti-Ki67 (1∶500, Novocastra, Newcastle, UK), rabbit anti-cleaved caspase-3 (1∶500, Cell Signaling Technology, Danvers, MA) and rabbit anti-TuJ1 (1∶1,000, Covance, Emeryville, CA). The Elite ABC kit (Vector Laboratories, Burlingame, CA) was used after biotin-conjugated secondary antibodies for signal amplification. For fluorescence immunostaining, avidin-Texas red (1∶1,000) and avidin-FITC (1∶1,000) were used for signal detection. For the cell cycle exit assay, sequential double immunostaining of BrdU and Ki67 was performed as previously described [30] with the following modifications: the sections were incubated with the antigen-retrieval solution containing 10 mM citric acid (pH 6.0) at 95°C for 10 min followed by 1 N HCl treatment at 45°C for 15 min. Rat anti-BrdU and mouse anti-Ki67 antibodies, biotinylated rabbit anti-rat and goat anti-mouse antibodies were used with the Elite ABC procedure followed by TSA-FITC (for detecting BrdU) and TSA-Cy3 (for detecting Ki67). Avdin-biotin blocking was performed after the first BrdU immunostaining by incubating the sections with solution A (1∶50, Vector laboratories) for 30 min and solution B (1∶50) for 30 min followed by 1% H_2_O_2_ treatment for 30 min. For triple immunostaining, the sections were sequentially immunostained for Ki67, TuJ1 and BrdU. The immunostaining of Ki67 and BrdU was as described above, and rabbit anti-TuJ1 antibody and goat ant-rabbit antibody conjugated with Alexa649 were used to detect TuJ1 signals in the triple immunostaining. The sections were counterstained with DAPI (1∶10,000). For some sections, after Ki67 and TuJ1 immunostaining, the brain sections were then processed for TUNEL staining using the *In Situ* Cell Death Detection kit (Roche, Indianapolis, IN). Before the TUNEL reaction mixture application, the slides were treated with 0.1 M glycine in PBS buffer for 30 min and then incubated with 0.1% Triton X-100 in 0.1% sodium citrate buffer at 4°C for 2 min.

### Image Analyses, Quantification and Statistics

Microscopic analysis was performed using Nikon E800M, Olympus BX63 and Leica DM6000B microscopes. Photomicrographs of X-gal stained embryos and dissected transgenic brains were taken by Nikon SMZ-1000 and NIS-element D3.0 software. Contrast and brightness adjustments for figures were made, and final plates were composed by using Adobe Photoshop CS (Adobe Systems, San Jose, CA). Forebrain sections, in which the borders of LGE, MGE and cortex were easy identified by morphology, were selected for quantification. Paired E12.5 brain sections of nCT and control T embryos from three independent littermates were analyzed for single PH3 and BrdU immunostaining. The numbers of PH3- and BrdU-positive cells in ventricular zone on left and right sides of brains were both calculated. Cell counting was performed under microscopy. After normalized with the lengths of ventricular zone or area of germinal zones (which were quantified by Image J software), the quantified data of PH3- and BrdU-positive cells in nCT brains were compared to that of control T littermate (PH3 cell counts) or T and C littermates (BrdU cell counts). For the cell cycle exit assay, telencephalic brain sections of 3–5 pairs of nCT, nC, T and WT embryos from four litters were analyzed. The numbers of BrdU+/Ki67− and total BrdU+ cells within 2 or 3 squares (50 µm×50 µm) of the cortex, LGE and MGE were counted in the comparable brain sections from each genotype. *Student*’*s t* test was used for statistical analysis.

## Results

### Generation of the LacZ^floxed^-Nolz-1^eGFP^ Transgenic Mice

**Figure 1:**
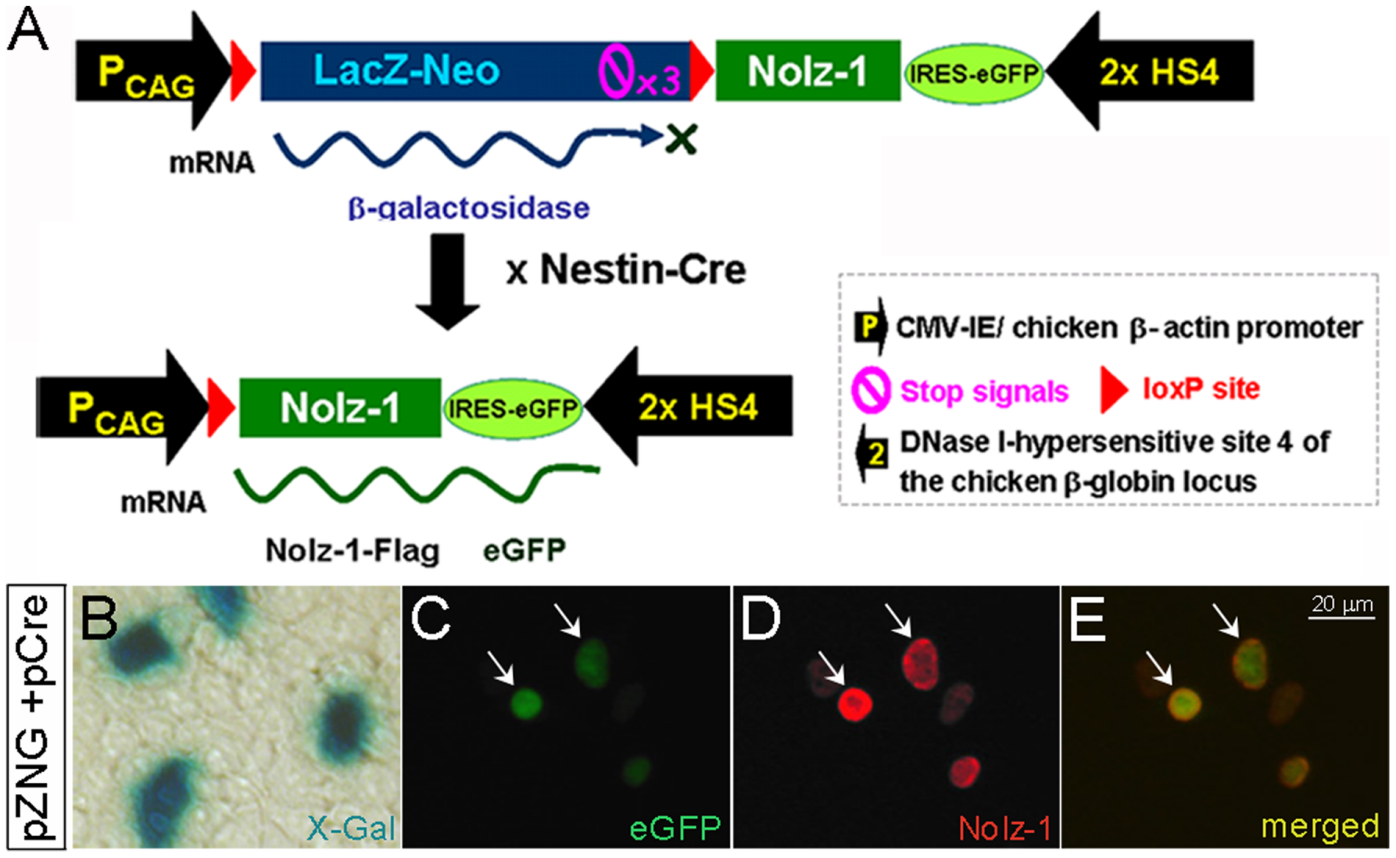
Generation of conditional *Nolz-1* transgenic mice and expression of transgenes *in vitro*. A: The strategy for generating nestin-Cre mediated Nolz-1^eGFP^ transgenic mice. B–E: Expressions of the transgenes LacZ (B), eGFP (C), Nolz-1 (D) were detected in the transfected HEK293T cells. Cells co-expressing eGFP (green, C) and Nolz-1 (red, D) were indicated by the arrows. E: Merged images of C and D.

We generated the transgenic mice carrying Cre-dependent *Nolz-1* allele. The transgenic construct contained a CMV enhancer/β-actin (CAG) promoter followed by the loxP-flanked LacZ-stop-Neo (LacZ-Neo) cassette which contained the β-galactosidase/neomycin resistance fusion gene and a triple polyA transcription termination (Stop) signal. Downstream of this cassette was the expression cassette of Nolz-1-Flag-IRES-eGFP-HS4, i.e., cDNAs encoding Flag-tagged *Nolz-1*, internal ribosomal entry site (IRES), enhanced green fluorescent protein (eGFP) reporter gene and two repeats of HS4 insulators [31] (Fig. 1A). The presence of an upstream “Stop” signal allowed the expression of β-galactosidase, but not the downstream Nolz-1 and eGFP. Upon the Cre-mediated DNA recombination, the loxP-flanked LacZ-Neo cassette was removed to permit the expression of Nolz-1 and eGFP by the CAG promoter (Fig. 1A).

Before performing the pronuclei microinjection in mice, we tested the expression of transgenic construct in HEK293T cells. Successful expressions of *LacZ*, Cre-mediated transgenic *Nolz-1* (*tNolz-1*) and *eGFP* were confirmed in transfected HEK293T cells (Fig. 1B–E).

The transgenic construct was microinjected into the female pronucleus of C57/BL6 mice for generating the LacZ^floxed^-Nolz-1^eGFP^ transgenic (Nolz-1 Tg) mice. The CAG promoter directed the transgenes expression presumably ubiquitously in the developing embryo. Among 23 lines of Nolz-1 Tg mice screened, #1, #3 and #9 were germ-line transmitted to F1 offspring. However, no transgenic F2 offspring was born from Nolz-1 Tg line #1. The X-gal staining analysis showed that the spatiotemporal expression pattern of *LacZ* in Nolz-1 Tg founder line #3 and #9 were different. *LacZ* expression was detected as early as embryonic day (E) 7.5 in Nolz-1 Tg line #9 (Fig. S1A, B). In contrast, *LacZ* expression was not observed until E13.5 in Nolz-1 Tg line #3 (data not shown). Although *LacZ* expression was detected in differentiated neurons in both line #3 and #9 Tg brains, *LacZ* expression was only detected in neural progenitor cells of the germinal zone in the brains of line #9 (data not shown).

### Transgenic Expression of *Nolz-1* in Neural Progenitors of the Nestin-Cre/LacZ^floxed^-Nolz-1^eGFP^ nCT Brain

**Figure 2:**
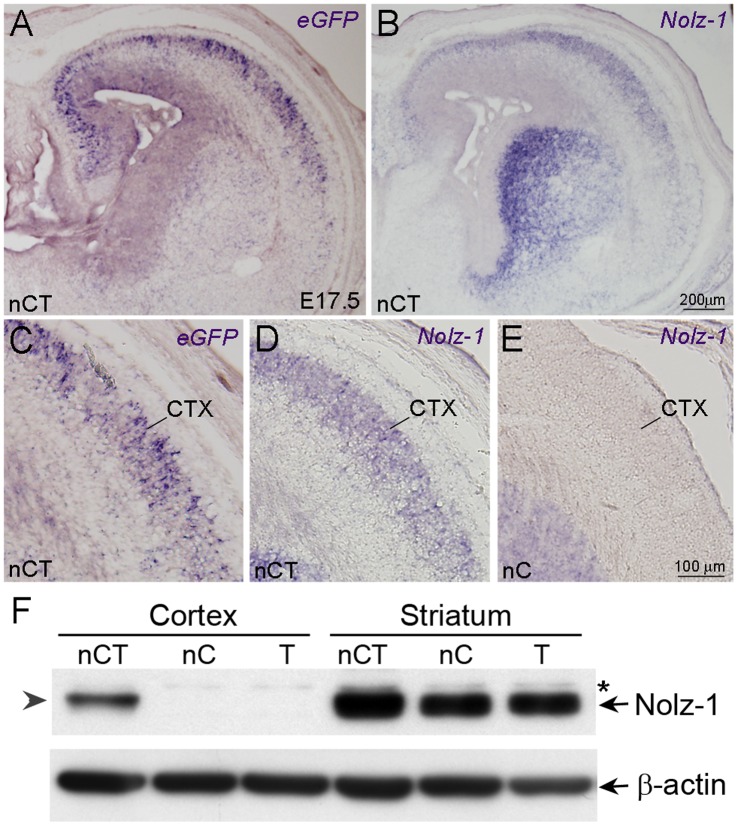
Ectopic expression of transgenic *Nolz-1* and *eGFP* in embryonic nCT brains. A–E: *In situ* hybridization demonstrates ectopic expression of *eGFP* (A, C) and *tNolz-1* (B, D) mRNAs in the cerebral cortex (CTX) of E17.5 nCT brain, but not in the control of nC brain (E). F: The Western blotting with anti-Nolz-1 antibody shows that the band of transgenic Nolz-1 protein (tNolz-1, arrowhead) was detected in the cortex of E16.5 nCT brain with size similar to that of endogenous striatal Nolz-1 protein (arrow) in the control nC and T brains. There was no detectable Nolz-1 protein in the cortex of the control nC or T brains. Asterisk indicates a non-specific band. Scale bars in B (for A, B), 200 µm, in E (for C–E), 100 µm.

**Figure 3:**
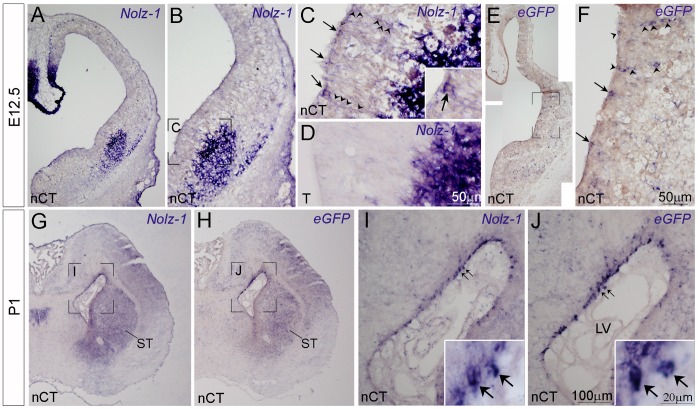
Ectopic expression of *tNolz-1* in telencephalic neural progenitor cells of the nCT brain. A–F: *Nolz-1* and eGFP mRNA-positive cells (arrows in C and F, respectively) were present in the ventricular zone of LGE in E12.5 nCT brain (A–C, E, F), but not in the ventricular zone of the control T brain (D). Arrowheads in C and F indicate *tNolz-1* and *eGFP* mRNA-positive cells lined up in radial lines. Ectopic *Nolz-1* mRNA-positive cells (I, inset) and *eGFP* mRNA-positive cells (J, inset) remained in the ventricular zone of lateral ventricle (LV) of nCT brain at postnatal day 1 (P1). B, C, F, I and J show the boxed regions at high magnification in A, B, E, G and H, respectively. Scale bars in D (for C, D) and in F, 50 µm, in I (for I, J), 100 µm, in inset of I (for insets of I, J), 20 µm.

The LacZ^floxed^-Nolz-1^eGFP^ Nolz-1 Tg #9 founder line was intercrossed with the nestin-Cre mice to express the transgenic *Nolz-1* (*tNolz-1*) in neural progenitor cells. Embryonic brains of the nestin-Cre;LacZ^floxed^-Nolz-1^eGFP^ (nestin-Cre;Nolz-1^eGFP^, nCT) and its control wild type (WT), nestin-Cre (nC) and LacZ^floxed^-Nolz-1^eGFP^ (T) littermates, were analyzed. Three lines of evidence confirmed the expression of *tNolz-1* in the nCT brain. First, the mRNAs of *tNolz-1* and *eGFP* transcribed by the transgenic CAG promoter were co-expressed in embryonic and neonatal nCT brains (Figs. 2A–E and 3). High levels of *tNolz-1* and *eGFP* mRNAs were co-expressed in the developing cortex where little endogenous *Nolz-1* was present (Fig. 2A–E) [11]. Second, consistent with the *in situ* hybridization analysis, the Western blot analysis showed that tNolz-1 protein expression was detected in E16.5 cerebral cortex of the nCT brain, but not in the cortex of the nC or the T control littermate brains (Fig. 2F). Third, *tNolz-1* and *eGFP* mRNAs were detected in the ventricular zone (VZ) of E11.5–E12.5 lateral ganglionic eminence (LGE, striatal primordium) where endogenous *Nolz-1* was absent (Fig. 3A–F) [11]. Note that, interestingly, some *tNolz-1* and *eGFP* mRNA-positive cells appeared to line up along a radial line in the germinal zone of LGE at E12.5 (Fig. 3C and F). Similar pattern of LacZ-positive cells along radial lines were also observed in the striatum and cortex of T brains of postnatal day 1 and three-weeks-old (Fig. S1C–E). It implicated that clones of post-mitotic striatal cells expressing the transgenes were undergoing radial migration in the germinal zone. Ectopic expression of *tNolz-1* and *eGFP* mRNA in neural progenitor cells of VZ persisted in neonatal nCT brains (Fig. 3G–J).

### Hypoplasia of Telencephalon in the nCT Double Transgenic Mice

**Figure 4:**
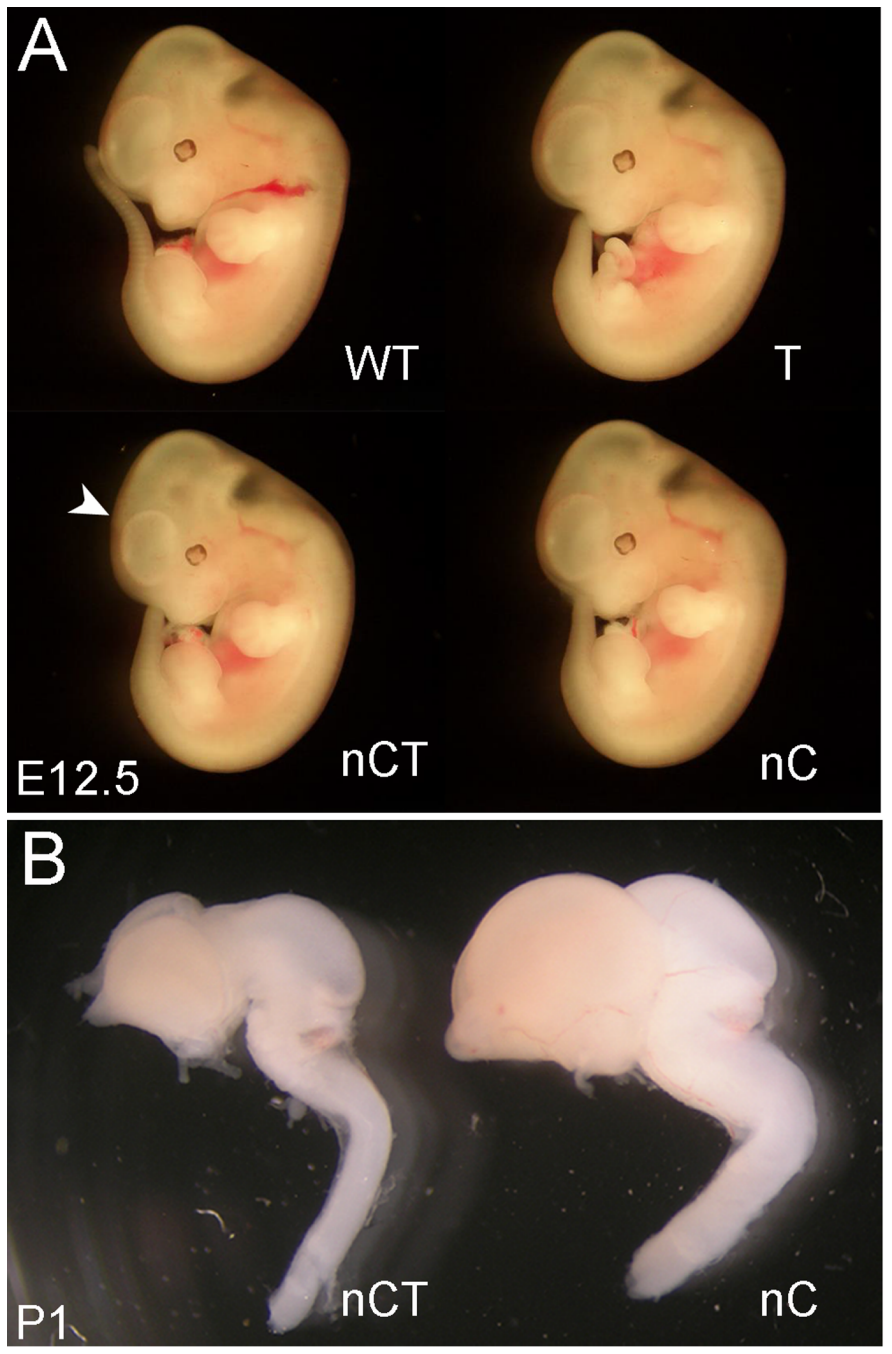
Hypoplasia phenotype of the nCT brain. A: Apparent hypoplasia of the nCT brain, particularly in the telencephalic region (arrowhead), was observed in E12.5 embryos B: Prominent hypoplasia was observed in postnatal day 1 (P1) nCT brain.

The analysis of the embryonic and neonatal nCT brains of line #9 showed that the head size was dramatically reduced in the nCT double transgenic mice (Fig. 4). Apparent hypoplasia of the telencephalic region was observed in E12.5 nCT embryo (Fig. 4A). A prominent hypoplasia of the nCT brain was also found one day after birth (Fig. 4B). The overall structure of the nCT brain, including the forebrain, midbrain and hindbrain, was not significantly changed, though the brain size was reduced compared to that of the control littermates (Fig. 4B).

### Reduction of Proliferative Neural Progenitor Cells in the nCT Brain

**Figure 5:**
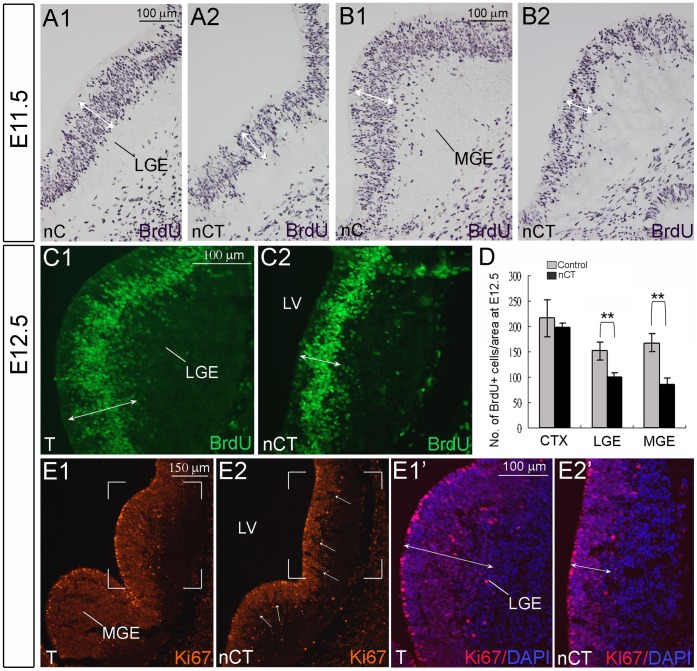
Reduction of proliferative neural progenitor cells in the nCT brain. A1-B2: BrdU pulse-labeled cells in the germinal zone were decreased in the LGE of E11.5 nCT brain (A2) compared to that in the nC brain (A1). Similar results were found in the MGE (B1, B2). C1–C2: Reduction of BrdU pulse-labeled cells in the germinal zone were also found in the LGE of E12.5 nCT brain (C2). The white double-headed arrows indicate the width of BrdU-positive germinal zone. D: Quantitative analysis of BrdU-positive cells in E12.5 forebrain regions. The graphical results represent mean ± SEM (control vs. nCT in CTX: 216±36 vs. 198±9; in LGE, 151±17 vs. 100±9; in MGE, 167±18 vs. 86±13). **, p<0.01, Student’s *t* test. E1–E2’: The Ki67-positive germinal zone was decreased in the nCT brain (E2) compared to the nC brain (E1). The bracketed regions in E1 and E2 are shown with DAPI counter staining at high magnification in E1’ and E2’, respectively. The white double-headed arrows indicate the width of Ki67-positive germinal zone. The arrows in E2 indicate radial lines that are devoid of Ki67-positive cells in the germinal zone of LGE and MGE. Scale bars in A1, B1, C1, E1’ for A1–2 B1–2, C1–2, E1’-2′, respectively, 100 µm; in E1 for E1–2, 150 µm.

The reduction of brain size might be resulted from decreased proliferation of neural progenitors and/or abnormal cell death. As early as E11.5–E12.5, reduced structures, including the cortical primordia, the LGE and the medial ganglionic eminence (MGE) accompanying with the enlarged ventricle were evident in the nCT brain (Fig. 5). Pulse labeling of proliferating cells with bromodeoxyuridine (BrdU) for 1 hr demonstrated a significant reduction of BrdU-positive cells in the germinal zones of LGE and MGE in E11.5 and E12.5 nCT forebrains (Fig. 5A1, A2, B1, B2, C1, C2). BrdU-positive cells were reduced by 34% and 48% in the LGE and MGE of E12.5 nCT forebrain, respectively, compared to control littermates (Fig. 5D). Consistent with the results of BrdU pulse-labeled cells, immunostaining of Ki67, a marker for proliferating cells, also showed significant reduction of Ki67-positive cells in E12.5 nCT forebrains (Fig. 5E1, E2, E1’, E2’). Of particular interest, there were several radial lines in which Ki67-positive proliferating cells were absent in the germinal zone of LGE and MGE of nCT brains (arrows, Fig. 5E2).

### Induction of Abnormal Programmed Cell Death in the nCT Brain

**Figure 6:**
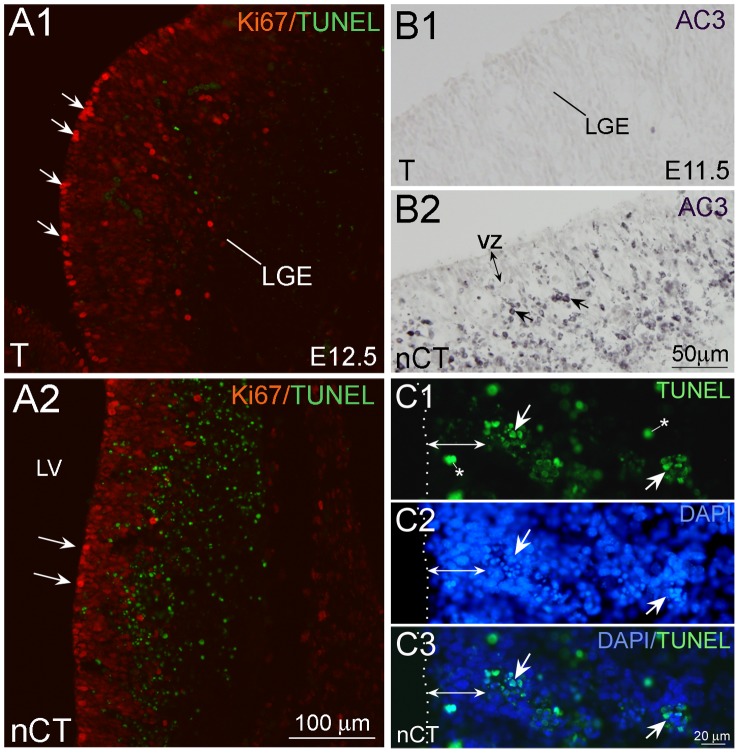
Induction of abnormal programmed cell death in the nCT brain. A1, A2: Many TUNEL-positive apoptotic cells were found in Ki67-positive germinal zone in the LGE of E12.5 nCT brain (A2). Few TUNEL-positive apoptotic cells were found in the control T brain (A1). Note that Ki67-positive cells lining along the lateral ventricle (arrows) were significantly decreased in the nCT brain (A1, A2). B1, B2: Activated caspase-3 (AC3)-positive apoptotic cells (arrows) were detected in the germinal zone of LGE in E11.5 nCT brain (B2), but not in that of the T brain (B1). Note that the majority of AC3-positive cells were present in the region beyond the ventricular zone of nCT brain (VZ, B2). C1–C3: Scattered TUNEL-positive cells (asterisk) and TUNEL-positive cell clusters (arrows) with condensed DAPI-positive nuclei were present in E12.5 nCT LGE. Note that few TUNEL-positive cells were present in the VZ. The double-headed arrows indicate the width of VZ. Scale bars in A2 (for A1, A2), 100 µm; B1 (for B1, B2), 50 µm; C3 (for C1–C3), 20 µm.

To further test if abnormal programmed cell death occurred in the nCT brain, we performed the terminal deoxynucleotidyl transferase dUTP nick end labeling (TUNEL) assay. TUNEL-positive apoptotic cells were increased in E12.5 nCT brains (Fig. 6A1, A2). Immunostaining of the activated caspase-3 (AC3) also showed increases of AC3-positive cells in E11.5 nCT brains (Fig. 6B1, B2). The abnormal cell death was found as early as E11.5, peaked at E12.5 and only a few AC3-positive cells were present in E15.5 nCT brains (Fig. S2). These findings indicated that ectopic expression of *tNolz-1* induced abnormal programmed cell death in neural progenitor cells. It was of particular interest that the majority of TUNLE-positive apoptotic cells were found in the SVZ, the region between the VZ and the MZ (Fig. 6A1, A2). Notably, distinct clusters of DAPI-positive condensed nuclei were present in the SVZ, and these clusters were positive for TUNLE signals (Fig. 6C1–C3), indicating that apoptotic cells were inclined to aggregate themselves to form apoptotic clusters.

### Reduction of Mitotic Neural Progenitors in the VZ of nCT Brain

**Figure 7:**
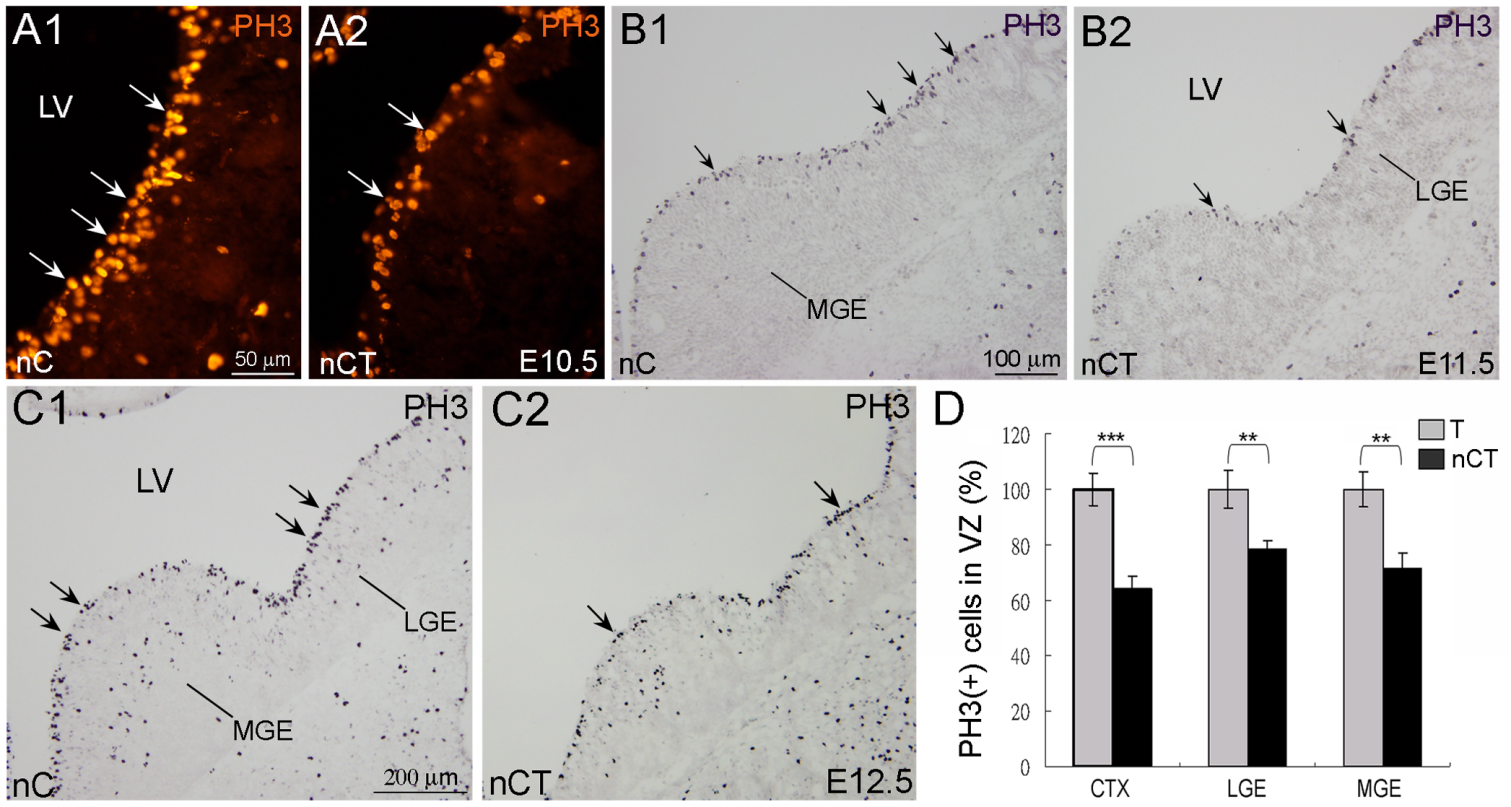
Reduction of mitotic neural progenitors in the ventricular zone of the nCT brain. A1–C2: PH3-positive cells (arrows) were decreased in the ventricular zone (VZ) of E10.5 subpallium (A2), E11.5 (B2) and E12.5 (C2) LGE and MGE of nCT brains compared to the control littermate of nC brains (A1, B1, C1). D: Quantitative analysis shows that the numbers of PH3-positive cells in the VZ of the cortex (CTX), LGE and MGE in E12.5 nCT brains were decreased compared to the T control brains. **, p<0.01, ***; p<0.001, Student’s *t* test. Scale bars in A1 (for A1, A2), 50 µm; B1 (for B1, B2), 100 µm; C1 (for C1, C2), 200 µm.

An interesting question was whether ectopic expression of *tNolz-1* in neural progenitors inhibited their proliferation. The concurrence of decreased proliferation and increased cell death in the SVZ of nCT brain made it difficult to resolve this issue. We then analyzed the mitotic cells in the VZ of nCT brain in which few TUNEL-positive apoptotic cells were found (Fig. 6C1–C3). Immunostaining of phospho-histone H3 (PH3), a mitotic marker, showed that PH3-positive cells undergoing the mitosis phase of cell cycle progression were lining along the wall of VZ (Fig. 7). PH3-positive cells were significantly decreased in the VZ of E10.5 subpallium, E11.5 and E12.5 LGE and MGE of nCT brains compared to the control brains (Fig. 7A1–C2). The quantitative analysis showed that PH3-positive cells in the VZ of cortical primordium, LGE and MGE in E12.5 nCT brains were decreased to 64±4.2%, 78±3.2% and 72±5.6% of the T control brains, respectively (Fig. 7D). Consistent with these findings, Ki67-positive proliferating cells lining along the surface of VZ were also decreased in the nCT brain (Fig. 6A1, A2). These results suggest that ectopic expression of *tNolz-1* in neural progenitors may inhibit proliferation of progenitor cells.

### Promotion of Cell Cycle Exit by *tNolz-1* in the nCT Telencephalon

**Figure 8:**
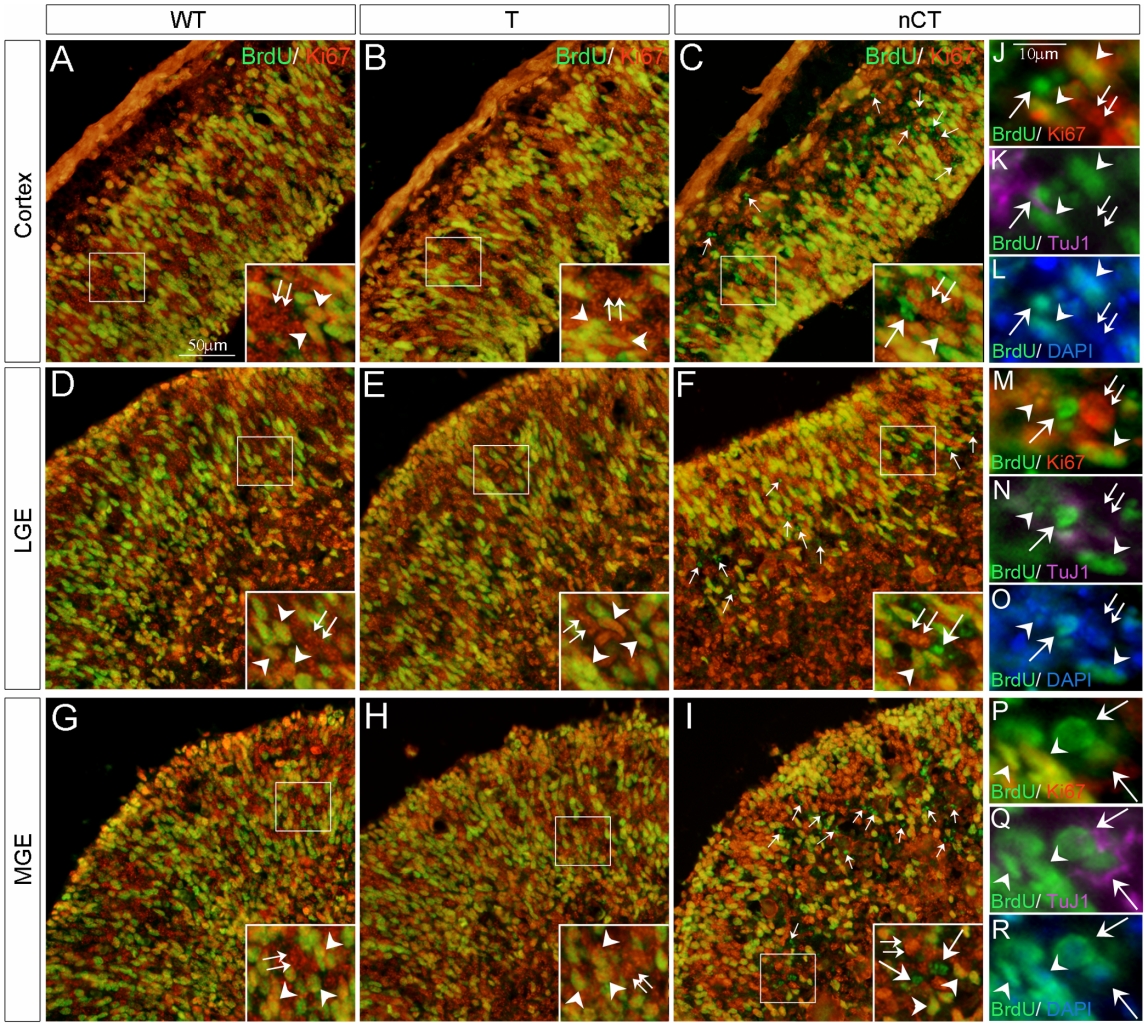
Promotion of cell cycle exit by *tNolz-1* in the nCT telencephalon. A–I: Double immunostaining of Ki67 (red) and BrdU (green) in E12.5 WT (A, D, G), T (B, E, H) and nCT (C, F, I) telencephalon. E12.5 brains were harvested 4 hr after pregnant mice were injected with BrdU. Many BrdU+/Ki67− cells (single arrows in C, F, I) were detected in the cortex, LGE and MGE of the nCT brain. Double arrows and arrowhead indicate BrdU?/Ki67+ cells and BrdU+/Ki67+ cells, respectively. Note that in the nCT brain (C, F, I), a few BrdU+/Ki67− cells (single arrows) appeared to line up along a radial line in the germinal zone of LGE as if they were derived from a clone of progenitors. J-R: Triple immunostaining of BrdU (green), Ki67 (red) and TuJ1 (purple). A few cells co-expressing BrdU and TuJ1 without Ki67 (single arrows) representing premature neurons were found in the germinal zone of cortex (J-L), LGE (M-O) and MGE (P-R) in the nCT brain. Double arrows indicate single Ki67-positive proliferative cells. Arrowhead indicates double BrdU− and Ki67-positive proliferative cells. Sections were counterstained with DAPI (blue). Scale bar in A (for A–I), 50 µm; in J (for J-R), 10 µm.

To further clarify the mechanism underlying *tNolz-1* inhibited proliferation of neural progenitor cells, we performed the cell cycle exit assay. Considering the severe programmed cell death in nCT brains, the BrdU-pulsing period was shortened to 4 hours. Double labeling of BrdU and Ki67 showed that many BrdU+/Ki67− cells were present in the germinal zone of cortex, LGE and MGE of nCT brains (arrows, Fig. 8C, F, I), compared to few BrdU+/Ki67− cells in the littermates control brains (Fig. 8A, B, D, E, G, H). The quantification analysis showed that the cell cycle exit index of the cortex, LGE and MGE of nCT brain were 8.7%, 8.5 and 10.6%, respectively; whereas the cell cycle exit index in the littermates control T, nC and WT brains were 0?0.5% (Table 1). Note that some BrdU+/Ki67− cells appeared to line up along a radial line in the germinal zone of LGE as if they were derived from a clone of progenitors. Notably, similar Ki67−negative radial-orientated domains were aforementioned (arrows, Fig. 5E2). Taken together with the finding of *tNolz-1* and *eGFP* mRNA-expressing cells along radial lines in the LGE of E12.5 nCT brain (Fig. 3C, F) these results suggest that *tNolz-1* over-expression in progenitor cells promotes cell cycle exit in the germinal zone of nCT telencephalon.

**Table 1:** Cell cycle exit index in E12.5 brains.

Region	Cell type	WT	nC	T	nCT
	1. BrdU+;Ki67−	0	0.1±0.1	0	2.2±0.1
**Cortex**	2. BrdU+;Ki67+	22.9±2.1	24.1±2.8	27.8±0.8	25.6±0.6
	**1/(1+2)**×**100% = cell cycle exit index**	**0%**	**0.4%**	**0%**	**8.70%**
	1. BrdU+;Ki67−	0	0.17±0.17	0	2.3±0.2
**LGE**	2. BrdU+;Ki67+	27.3±1.2	26±2.4	28±0.8	28±0.5
	**1/(1+2)**×**100% = cell cycle exit index**	**0%**	**0.5%**	**0%**	**8.50%**
	1. BrdU+;Ki67−	0	0	0	2.8±0.4
**MGE**	2. BrdU+;Ki67+	26.8±2.6	26.8±0.2	30.7±1.6	26.5±1.3
	**1/(1+2)**×**100% = cell cycle exit index**	**0%**	**0%**	**0%**	**10.60%**

WT: wild type brain; nC: nestin-Cre brain; T: LacZ^floxed^-Nolz-1^eGFP^ brain; nCT: nestin-Cre;LacZ^floxed^-Nolz-1^eGFP^ brain; LGE, lateral ganglionic eminence; MGE: medial ganglionic eminence.

### Precocious Neuronal Differentiation in the nCT Brain

Because endogenous *Nozl-1* was expressed in differentiating neurons, another interesting question was whether ectopic expression of *tNolz-1* in neural progenitors could promote neuronal differentiation. In the aforementioned cell cycle exit assay, we also performed triple immunostaining of BrdU, Ki67 and the neuron-specific class III beta-tubulin (TuJ1), a marker for early differentiating neurons [32]. Interestingly, a few cells co-expressing BrdU and TuJ1 without Ki67 that represented precociously differentiating neurons prematurely exiting cell cycle were found in the germinal zone of nCT telencephalon. (Fig. 8J–R).

**Figure 9:**
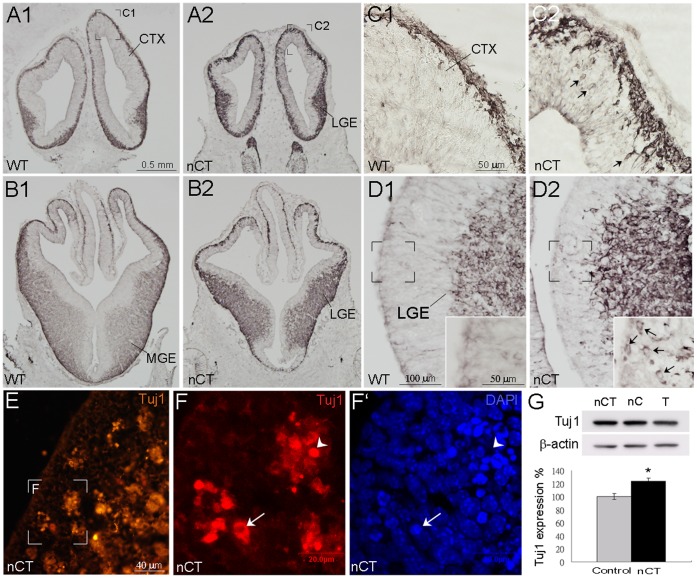
Ectopic and precocious TuJ1 expression in the proliferative ventricular zone of E12.5 nCT brain. A1-D2: TuJ1 immunoreactivity was higher in the nCT forebrain (A2, B2, C2) than that in the control forebrain (A1, B1, D1). Ectopic and precocious TuJ1-positive neurons were found in the TuJ1-poor ventricular zone of the cerebral cortex (C2, arrows) and LGE (D2, arrows). The bracketed regions in D1 and D2 are shown at high magnification in the insets of D1 and D2, respectively. E: TuJ1-positive cell clusters are present in the germinal zone of the nCT brain. The bracketed region in E is shown at high magnification in F with confocal images. F, F’: TuJ1-positive cell clusters contain apoptotic nuclei with condensed DAPI staining. The arrow indicates a TuJ1-positive neuron (F) whose nucleus was presumably under the process of DNA condensation as implicated by the condensed DAPI staining (F’). The arrowhead indicates a presumed degrading cell with condensed TuJ1 immunoreactivity without apparent DAPI staining. G: Western blotting and quantification of TuJ1 protein expression in E12.5–13.5 cortex. TuJ1 expression in nCT cortex is 123±4.5% of control littermates (p<0.05). Scale bars in A1 (for A1, A2, B1, B2), 0.5 µm; C1 (for C1, C2) and in inset of D1 (for insets of D1, D2), 50 µm; D1 (for D1, D2), 100 µm; E, 40 µm; F–F’, 20 µm.

Despite the abnormal cell death in the nCT brain, TuJ1 immunostaining showed an apparently general increase of TuJ1 immunoreactivity in E12.5 nCT brain compared to that of the control brain (Fig. 9A1–D2). The Western blotting further confirmed increases of TuJ1 expression in E12.5–E13.5 nCT cortical primordia compared to the control littermates (123±4.5%, p<0.05; Fig. 9G).

TuJ1 immunostaining also showed that TuJ1-positive MZ appeared to expand toward the germinal zone (Fig. 9C1, C2, D1, D2). Scattered TuJ1-positive neurons were ectopically found in the VZ of nCT telencephalon, but not in the control telencephalon (Fig. 9D1, D2). Moreover, cell clusters with enhanced TuJ1 immunostaining were present in the germinal zone of nCT brain (Fig. 9E, F). These strong TuJ1-positive clusters contained condensed DAPI-positive nuclei, which was the sign of initiating apoptotic process (Fig. 9F’).

**Figure 10:**
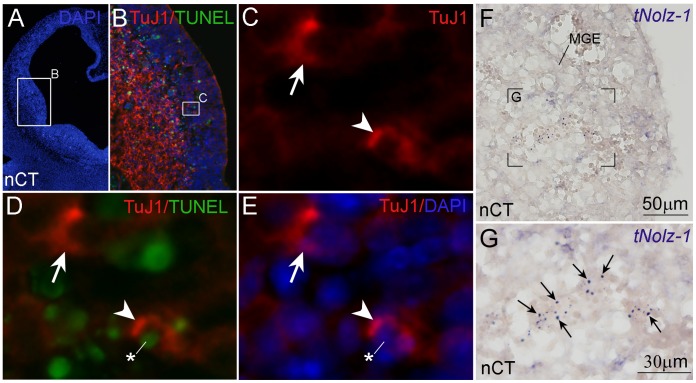
Double labeling of TuJ1 and TUNEL and *tNolz-1* expression in apoptotic cell clusters of the germinal zone in E12.5 nCT forebrain. A–E : Double labeling of TuJ1 and TUNEL. A: DAPI staining. B, D: Merged images of TuJ1 (red) and TUNEL (green) in the LGE germinal zone. E: Merged images of TuJ1 (red) and DAPI (blue). B and C–E show the boxed regions at high magnification in A and B, respectively. D: TUNEL-positive signals (asterisk) were detected in some TuJ1-positive cells (arrowhead) ectopically localized in the germinal zone. The arrow indicates a single TuJ1-positive cell without TUNEL signal. F, G: *tNolz-1* mRNA was detected as presumably inclusion bodies (arrows, G) in some apoptotic cell clusters in the MGE of nCT brain.

Indeed, double-labeling of TuJ1 and TUNEL confirmed co-localization of TuJ1 and TUNEL signals in some ectopic TuJ1-positive cells in the germinal zone of nCT forebrain (arrowhead, Fig. 10), though a few other TuJ1-positive germinal cells were negative for TUNEL signals (arrow, Fig. 10D). Interestingly, in some apoptotic cell clusters which appeared to line up as a radial line, *tNolz-1* mRNA expression was detected as condensed particles (Fig. 10F, G; arrows). Taken together, these results suggest that inappropriate precocious differentiation triggered by *tNolz1* in some neural progenitor cells may lead to abnormal apoptosis in the nCT brain.

### Induction of Abnormal Apoptosis by Over-expression of *Nolz-1* in N18, ST14A and N2A Neural Cells

**Figure 11:**
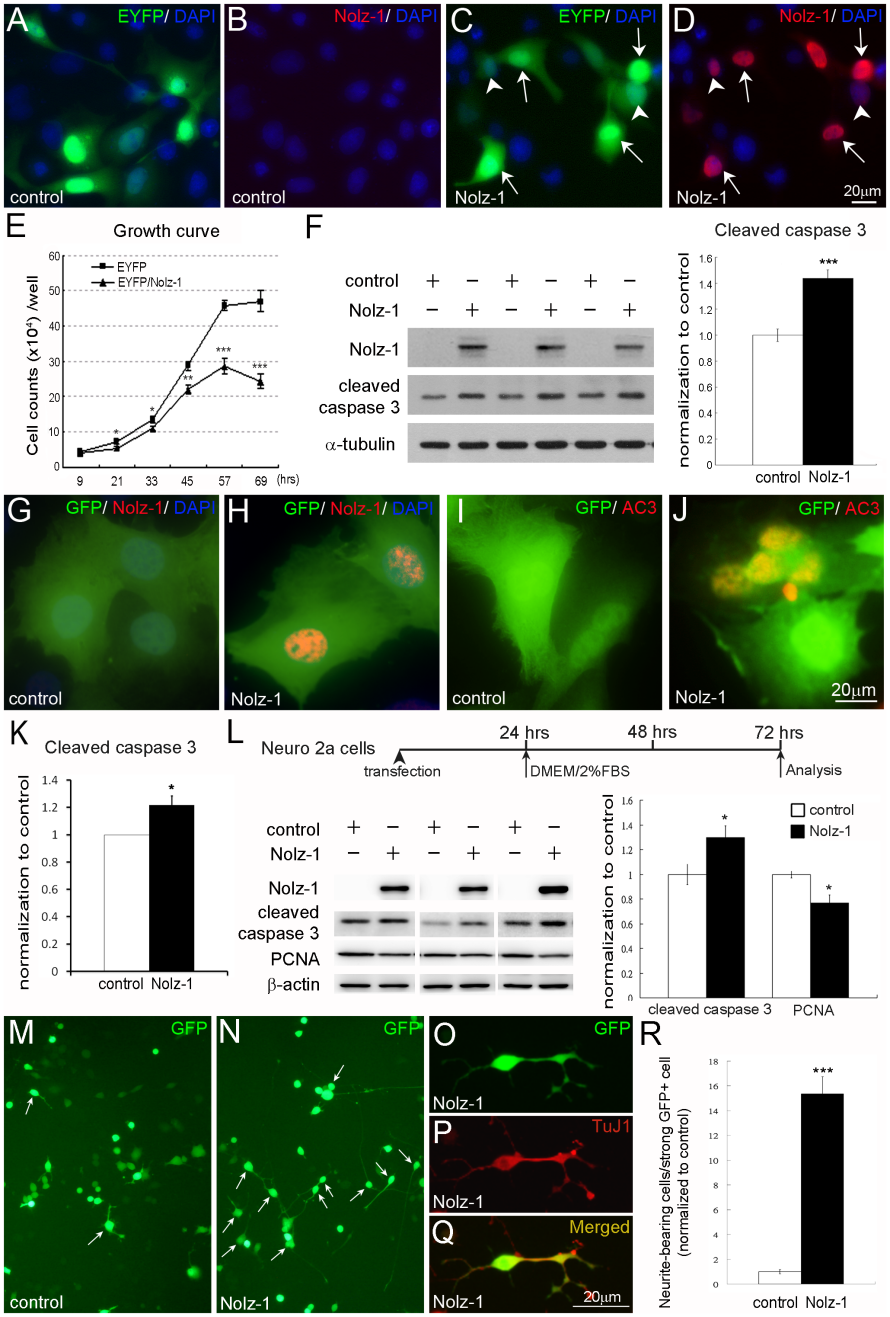
Induction of abnormal apoptosis by over-expression of *Nolz-1* in N18, ST14A and N2A neural cells. A–F: *Nolz-1* over-expression led to an increase of cell death in N18 cells. N18 cells were co-transfected with pNolz-1 and pEYFP constructs. Co-localization of Nolz-1 protein (red, B) and GFP (green) were found in all GFP-positive N18 cells (arrows, C and D), but not in GFP-positive cells of mock control (A, B). E: The growth curve analysis of *Nolz-1*-transfected N18 cells. The number of *Nolz-1*-transfected N18 cells was progressively decreased with increasing culture times. F: Western blotting of cleaved caspase 3 shows a 1.4-fold increase of cleaved caspase 3 in N18 cells 24 hr after transfection. G,-J: Transfection of *Nolz-1* (red, H) induced active-caspase 3 (AC3, red, J) in ST14A cells 24 hr after transfection. K: Quantitative analysis of AC3-positive ST14A cells with *Nolz-1* over-expression. L: Induction of cleaved caspase 3 and reduction of PCNA in *Nolz-1*-transfeceted N2A cells. Upper panel shows the experimental procedure. The Western blotting shows increases and decreases of cleaved caspase 3 and PCNA, respectively, in *Nolz-1*-transfected N2A cells M-Q: Promotion of neuronal differentiation by *Nolz-1* over-expression in N2A cells. N2A cells were co-transfected with *Nolz-1* and GFP. *Nolz-1* over-expression induced a 15-fold increase of the number of neurite-bearing neurons (arrows, N) compared to the mock transfected group (M) 72 hr after transfection. O–Q: *Nolz-1*-transfected cells expressed the neuronal differentiation marker of TuJ1. R: Quantitative analysis of neurite-bearing cells with *Nolz-1* over-expression. *, p<0.05; **, p<0.01; ***, p<0.001, Student’s *t* test. Scale bars in D (for A–D), in J (for G–J) and in Q (for O–Q), 20 µm.

Our transgenic mouse study indicated that *tNozl-1* promoted cell cycle exit, premature neuronal differentiation and induced abnormal apoptosis of neural progenitor cells in developing telencephalon. To further validate these findings at the cellular level, we carried out *Nolz-1* over-expression study in three different neural cells lines, including the mouse neuroblastoma N18, neuro-2a (N2A) cells and the immortalized ST14A cells derived from the rat striatal primordia (Fig. 11) [26]–[28]. For monitoring the transfected cells, the expression plasmids containing a dual CAG promoter driving Nolz-1 and EYFP/GFP were used. Co-localization of Nolz-1 protein and EYFP/GFP were found in all EYFP/GFP-positive cells, but not in EYFP/GFP-positive cells of mock control (Fig. 11A–D, G, H). The growth curve analysis of N18 cells showed that the number of *Nolz-1*-transfected N18 cells was decreased with increasing times in culture (Fig. 11E). A 1.4-fold increase of cleaved caspase 3 expression was observed 24 hr after *Nolz-1* transfection (143.6±3.4%, n = 6, p<0.001; Fig. 11F), indicating abnormal apoptosis induced by over-expression of *Nolz-1*. Similar effect of *Nolz-1* over-expression in induction of apoptotic cell death was also observed in ST14A and N2A cells (Fig. 11I–L). Immunostaining of active caspase-3 (AC3) showed a 1.2-fold increase of AC3-positive ST14A cells 24 hr after *Nolz-1* transfection (121.9±5.4%, n = 3, p<0.05; Fig. 11K). In N2A cells, a 1.3-fold increase of cleaved caspase 3 was found 72 hr after *Nolz-1* transfection (129.7±9.5%, n = 3, p<0.05; Fig. 11L).

### Reduction of Cell Proliferation and Promotion of Neuronal Differentiation by *Nolz-1* Over-expression in N2A Neural Cells

In addition to abnormal apoptosis**,** *Nolz-1* over-expression also resulted in reduction of PCNA, a proliferating cell marker, to 76.8±6.4% of the mock control in N2A cells (n = 3, p<0.05; Fig. 11L). Interestingly, accompanying with the decrease of cell proliferation, many *Nolz-1*-transfected N2A cells morphologically differentiated into TuJ1-positive neurite-bearing neurons (Fig. 11M–Q). Significant numbers of *Nolz-1* transfected cells extended longer TuJ1-positive neurites compared to the mock control. The quantitative analysis indicated that *Nolz-1* over-expression induced a 15-fold increase of the number of neurite-bearing neurons compared to mock control (15.4±1.38, n = 5, p<0.001; Fig. 11R).

Taken together with these results, it indicated that *Nolz-1* over-expression induced abnormal apoptosis, suppressed cell proliferation and promoted neuronal differentiation in neural cell culture. These findings in cell culture *in vitro* are in good agreement with the findings in *Nolz-1* Tg brains *in vivo*.

## Discussion

In the present study, we investigated the function of *Nolz-1* in developing telencephalon using conditional transgenic mouse approach. Because *Nolz-1* is not expressed by proliferative neural progenitors in the germinal zone, we studied the effects of ectopic expression of *Nolz-1* in neural progenitors using the nestin-Cre driver mice. Nestin-Cre dependent expression of *tNolz-1* in neural progenitors of forebrain resulted in decreases of neural progenitors. There are two possibilities to account for the reduction of neural progenitors. These two possibilities are not exclusive to each other. One is that ectopic expression of *tNolz-1* induces apoptosis of progenitor cells. Indeed, TUNEL-positive and activated caspase-3-positive apoptotic cells were found in the germinal zone of the nCT brain. The other possibility is that ectopic expression of *tNolz-1* promotes precocious neuronal differentiation, which in turn leads to depletion of progenitor pools. Consistent with this possibility, promotion of cell cycle exit and enhanced ectopic TuJ1 expression was found in the germinal zone of nCT brain. Notably, apoptotic cell clusters containing strong TuJ1-positive signals were found in the germinal zone of SVZ, suggesting that inappropriate premature differentiation of progenitors may cause abnormal cell death in some progenitor cells. In other progenitors, *tNolz-1*-promoted differentiating cells may survive, because there was a general increase of TuJ1 immunoreactivity in the nCT brain.

The transgenic expression of *Nolz-1* was driven by the CAG promoter that is likely to have different levels of activity in neural progenitor populations. It is plausible that the expression level of *tNolz-1* may be varied in neural progenitors of the nCT brain. A high level of *tNolz-1* may promote aberrant differentiation which eventually causes cell death, whereas a low level of *tNolz-1* may promote neuronal differentiation of progenitor cells without triggering apoptosis. If so, it implies that the physiological level of *Nolz-1* expression must be under rigorous control during neurogenesis. Consistent with this hypothesis, as post-mitotic *Nolz-1*-expressiing neurons migrate from the SVZ to the differentiated MZ, the expression level of *Nolz-1* goes through a transition from the high level in the SVZ to the low level in the mantle zone [10], [11].

### Aberrant Proliferation and Differentiation of Neural Progenitors Induced by Transgenic *Nolz-1*

The finding that PH3-positive mitotic cells were reduced in the ventricular zone in which few apoptotic cells were present suggests that transgenic *Nolz-1* may negatively regulate proliferation of neural progenitors. Analyses of *tNolz-1* and *eGFP* transgene expression showed that there were some *tNolz-1*- and *eGFP*-positive cells lining up alone radial lines as if they were derived from a clone (Fig. 3C, F). This radial lines pattern was also observed in single Ki67 immunostaining and double BrdU/Ki67 immunostaining in the cell cycle exit assay (Fig. 8). These data provide evidence supporting that the promotion of cell cycle exit/inhibition of cell proliferation in the germinal zone of telencephalic regions were associated with transgenic *Nolz-1* expression. Moreover, some ectopic TuJ1 expression in the germinal zone was observed to extend along radial lines in nCT brains, particular in the cortex (Fig. 9C2). Notably, strong ectopic TuJ1 expression was more prominently found in many apoptotic cell clusters in the germinal zone of MGE where transgenic *Nolz-1* mRNA was localized as condensed particles. Furthermore, triple immunostaining showed that a few cells co-expressing BrdU and TuJ1 without Ki67 which represented precociously differentiating neurons prematurely exiting cell cycle were found in the germinal zone of nCT telencephalon. These findings support the conclusion that transgenic *Nolz-1* not only promotes cell cycle exit/inhibits cell proliferation, but also promotes premature neuronal differentiation.

In addition to the cells lining up as radial lines, *tNolz-1* and *eGFP* mRNAs were also observed in scattered cells in the germinal zone of telencephalic nCT brains. This may explain the presence of some scattered BrdU+/Ki67− cells that were found in the cell cycle exit assay and the widely reduction of PH3- and Ki67-positive proliferating cells. Other possible causes of the widely reduction of proliferating cells may be non-cell autonomous effects of apoptosis induced by *Nolz-1* over-expression. Further study is required to elucidate these possibilities.

In our study, the findings of both *tNolz-1/eGFP* mRNA-expressing cells and BrdU+/Ki67− cells lining along radial lines in the LGE of E12.5 nCT brain suggest a cell-autonomous effect of *tNolz-1* over-expression in promoting cell cycle exit/inhibiting proliferation of progenitor cells in the germinal zone of nCT telencephalon. Induction of premature neuronal differentiation may be a consequence of promoting cell cycle exit by *tNolz-1* cell-autonomously. Given the induction of differentiation of N2A cells into TuJ1-positive neurite-bearing neurons by *Nolz-1* over-expression, the premature neuronal differentiation phenotype observed in telencephalic regions of nCT brains may be caused by a cell-autonomous effect of ectopic expression of *tNozl-1* in neural progenitor cells.

Notably, unlike the restricted expression of *Nolz-1* to post-mitotic neurons in developing striatum *in?vivo* [11], [14], it has been reported that *Nolz-1* is expressed in proliferative neural progenitors in neurosphere culture in which over-expression of *hNolz-1* results in increases of cell cycle exit [10]. On the other hand, functional studies of elbow (*elB*) and no ocelli (*noc*), two *Drosophila* homologous genes of *Nolz-1*, indicate regulatory control of cell proliferation by *elB* and *noc* [33], [34]. In the eye-antenna imaginal discs, which give rise to the adult head, eye and antenna, *elB* and *noc* are expressed in the proliferative region. In region anterior to the morphogenetic furrow, the *elB-noc* gene complex attenuates Notch-induced cell proliferation. Loss of *elB* and *noc* activities induces overgrowth of the head capsule [34]. Conversely, over-expression of *elB* by *eyeless*-GAL4 in proliferating cells of eye imaginal discs causes reduction of eye size. The small eye phenotype can be rescued by co-expression of the positive cell cycle regulator, *cycE* [33]. However, no significant phenotype is observed when *elB* is over-expressed in post-mitotic cells of the imaginal discs [33]. These findings implicate that *elB* acts as a negative cell cycle regulator in proliferating cells of *Drosophila* eye imaginal discs.

Our present study and previous studies from other groups suggest that *Nolz-1* is involved in regulation of cell proliferation. In N2A cells, over-expression of *Nolz-1* resulted in decreased PCNA proliferative signals accompanying with a prominent neuronal differentiation. Previous study has shown that proliferation of NIH3T3-A14 cells is regulated by cAMP, because the cells are arrested in G1 phase 24 and 48 hr after the forskolin/IBMX treatment. Interestingly, *Nolz-1* is induced 2 hr after forskolin/IBMX treatment, but not at other time points [35]. This suggests a transient role of *Nolz-1* in regulating proliferation of NIH3T3-A14 cells. This *Nolz-1* induction is MAPK-independent, because *Nolz-1* expression is not affected by the MEK-inhibitor U0126 [35].

Our study also suggests that *Nolz-1* promotes neuronal differentiation, because transgenic expression of *Nolz-1* in neural progenitors enhanced TuJ1 expression in the germinal zone of nCT brain. Increases of neuronal, but not glial, differentiation by over-expression of *hNolz-1* have previously been shown in a cell culture study [10].

### Abnormal Apoptosis Induced by Transgenic *Nolz-1*

Our present study indicates that transgenic ectopic expression of *Nolz-1* in neural progenitors induced abnormal apoptosis in the nCT brain. Consistent with our findings, previous study has shown that Hb9 promoter-driven over-expression of chicken homologue of *Nolz-1* in newly differentiating motor neurons induces abnormal apoptosis in the developing spinal cord [9]. These results suggest that constitutive expression of high levels of *Nolz-1* may eventually lead to abnormal cell death. In our study, over-expression of *Nolz-1* in three neural cell lines of different origins all increased apoptotic cell death. Taken together with these findings, over-expression of *Nolz-1* at high levels may have a common effect leading to programmed cell death in progenitor cells and differentiating neurons.

Previous studies have shown that the zebrafish and chick homologues of *Nolz-1* function as transcription repressors [4], [5], [7], [9]. The transcription repressor activity of zebrafish *Nlz1* is dependent on binding to groucho and class I histone deacetylases (HDACs), HDAC1 and HDAC2 [7]. During embryogenesis, HDAC1 and HDAC2 are expressed in neural stem cells/progenitors. Interestingly, aberrant apoptotic cell death and reduction of brain size, the phenotypes that were observed in the nCT brain, were also observed in HDAC1;HDAC2 double mutant mice [36]. Over-expression of *Nolz-1* may act as gain-of–function by chelating HDAC1 and HDAC2 from other genes that they regulated, which may then consequently induces abnormal programmed cell death.

The hypoplasia phenotype occurred all over the nCT brain, including the forebrain, midbrain and hindbrain without changing the overall structure of the nCT brain, though the brain size was reduced compared to that of the control littermates. Similar effects of *tNolz-1* on promoting cell cycle exit/inhibiting cell proliferation and inducing apoptotic cell death may also occurred in the regions of nCT brain other than telencephalon. It is likely that the induction of cell death by over-expression of *tNolz-1* occurs in diverse types of neural progenitors, because active caspase 3-positve cells were detected in several regions of the nCT brain. Indeed, in agreement with this finding, we found that *Nolz-1*-induced apoptosis occurred in three lines of neural cells *in vitro*. It is notable that in the hindbrain region where endogenous *Nolz-1* is also expressed [29], we also observed *tNolz-1*-positive apoptotic cell clusters and ectopically expressed TuJ-1 in the germinal zone of nCT hindbrain (data not shown). These results implicate that *tNolz-1* over-expression-mediated promoting cell cycle exit and premature neuronal differentiation might also occur in the hindbrain region of nCT brain.

## Supporting Information

Figure S1 **Expression of LacZ transgene in developing transgenic mouse embryos and brains.** A, B: X-gal positive signals were detected in the line #9 *Nolz-1* Tg embryo at E7.5 (A), but not in the control wild type embryo (B) . C–E: In the neonatal (P1) and three-week old (3 wk) transgenic brains, many X-gal positive cells were detected in the striatum (ST; C, D) and cerebral cortex (CTX; E).
(TIF)

Figure S2 **Immunostaining of active caspase 3 in E15.5 nCT telencephlon.** Comparing to E12.5, there were only a few active caspase 3 (AC3)-positive cells (arrows) in the cortex and striatum (ST) of nCT brain at E15.5. AC3-positive cells were nearly absent in the cortex and ST of nC control brain.
(TIF)
