# Supplementary figures and images for: Ectopic Expression of Nolz-1 in Neural Progenitors Promotes Cell Cycle Exit/Premature Neuronal Differentiation Accompanying with Abnormal Apoptosis in the Developing Mouse Telencephalon
Source: PLoS One. 2013 Sep 20;8(9):e74975. doi: 10.1371/journal.pone.0074975 (PMC3779228; doi:10.1371/journal.pone.0074975)

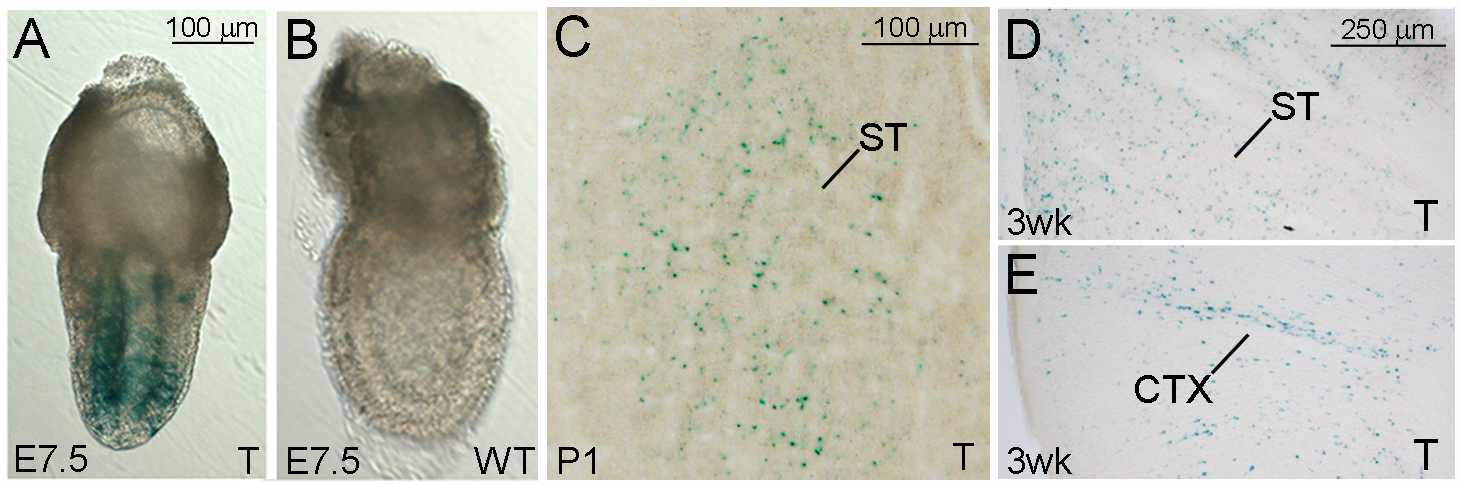

Supplement: Figure S1 — Expression of LacZ transgene in developing transgenic mouse embryos and brains. A, B: X-gal positive signals were detected in the line #9 Nolz-1 Tg embryo at E7.5 (A), but not in the control wild type embryo (B) . C–E: In the neonatal (P1) and three-week old (3 wk) transgenic brains, many X-gal positive cells were detected in the striatum (ST; C, D) and cerebral cortex (CTX; E). (TIF) [file pone.0074975.s001.tif]

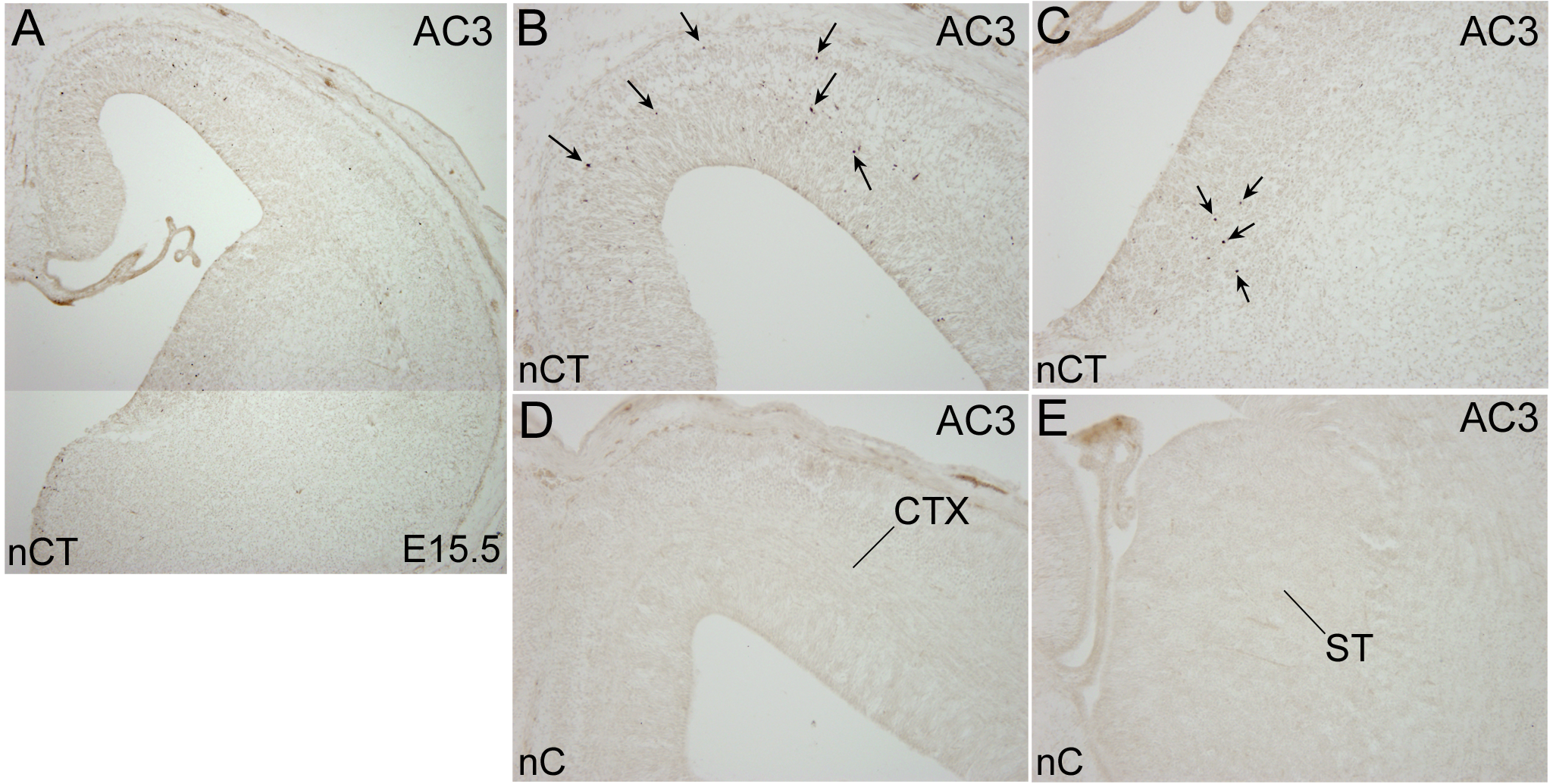

Supplement: Figure S2 — Immunostaining of active caspase 3 in E15.5 nCT telencephlon. Comparing to E12.5, there were only a few active caspase 3 (AC3)-positive cells (arrows) in the cortex and striatum (ST) of nCT brain at E15.5. AC3-positive cells were nearly absent in the cortex and ST of nC control brain. (TIF) [file pone.0074975.s002.tif]
